# A critical look at the Portuguese public–private partnerships in healthcare

**DOI:** 10.1002/hpm.3084

**Published:** 2020-10-04

**Authors:** Miguel A. Pereira, Diogo C. Ferreira, Rui C. Marques

**Affiliations:** ^1^ CEG‐IST Instituto Superior Técnico Universidade de Lisboa Lisboa Portugal; ^2^ CERIS Instituto Superior Técnico Universidade de Lisboa Lisboa Portugal

**Keywords:** contracts, National Health Service, Portugal, public–private partnerships

## Abstract

The pre‐conceived idea that contracts in a public–private partnership (PPP) regime, in healthcare or in any other economic sector, are, as a rule, ruinous and appealing for only a share of the stakeholders, lacks a solid basis that confirms it. This idea, outset and nurtured by the media, has been instigating the distrust of the users who, in turn, demand a more rigorous and efficient utilisation of public resources. Being Portugal in the top of countries that resort to PPPs, it is urgent to inquire if its respective contracts originated an inefficient and ineffective management of resources. It is precisely this discussion that we address in this paper, focusing our efforts in the Portuguese healthcare sector.

## 1 INTRODUCTION

The concept of public–private partnership (PPP) has been broadly discussed in several sectors, although not many are aware of its true potential and flaws. The pre‐conceived idea that this type of partnerships translates into ruinous contracts for the public treasury has its genesis on the inability of the State. First, the State does not properly avail the competitive potential of its strategic private partner, as well as the latter's most efficient capabilities in risk management. Second, the State reduces its bargaining power in face of its private counterpart, which results in a weak contract management and, consequently, numerous renegotiations that frequently damage the former, since they require rewards to the private party. Additionally, the optimism biasing effect, which results from a too high demand over supply, generates the same rewards.

Still, there are plenty of reasons to conduct such a partnership.^1^ It is widely known that both the deadlines and the budgets of public works frequently slip to values quite above the ones initially expected. Considering that private parties are payed a fixed price and they seek to maximise profit, the danger of slippage is minimised if the work is awarded to them. This way, it is possible to apply innovative solutions, ensure an active and dynamic management of infrastructures and services, and warrant a more rigorous selection of projects.^2^

In the case of healthcare, for the sake of its social relevance, it is important to discuss this topic from the perspective of the National Health Service's (SNS, from the Portuguese abbreviation of *Serviço Nacional de Saúde*) financial–economic sustainability. Several models have been adopted nationally and internationally, differing essentially at the level of integration of the private partner in the value chain.^3^ Currently, in Portugal, the management of the infrastructures and the clinical services is assigned to two private partners, financed according to the availability of each service and the number of treated users. Despite the sensitivity surrounding the assignment of clinical management to the private sector, this model is the only one in operation in new or replacement hospitals, even though it has been noted that this attribution should only be made if a hospital's technology of production does not show significant variations over time. However, this model has ceased and gave place to a more vulgar model at an international level—only the management of the infrastructure is a responsibility of the private partner—which is in line with the fast‐paced technological evolution in health. Until now, no hospital has been built in Portugal under this assumption. Furthermore, this is a model that will, indeed, allow the reduction in the number of potential renegotiations. Note that PPPs are not necessarily damaging contracts if they are well managed, formulated and regulated, preventing many renegotiations and an unbalanced distribution of risk.

The aim of this paper is to discuss whether the healthcare PPP contracts in Portugal yielded value gains to the State or originated an inefficient and ineffective management of resources.

## 2 PPPs IN THE HEALTHCARE SECTOR

### 2.1 Background

When providing social services, as is the case of healthcare, where the public sector is predominant, it is natural to think that the private sector should be used instead of the public one if it has some sort of advantage. Nonetheless, if it were possible to create a contract in which the frontiers between the responsibilities of each contractor were well defined, it would be advantageous to create a PPP whenever the costs of the private sector were inferior to the costs of its public counterpart in a given function.^4^ Healthcare PPPs rise as an alternative solution due to budgeting constraints in the public sector.^5^ The introduction of private management mechanisms in public sector hospitals is not uncommon,^3^, ^6^ since it represents a way of liberalising the sector. In the case of PPPs, the clinical and/or infrastructure management is a responsibility of the private sector, and the user simply needs to pay user charges, as if it were a public hospital.

According to Martimort and Pouyet,^7^ the main reason for considering a PPP contract regarding the construction and exploration of clinical activities is the existence of economies of scope and not the public or private nature, highlighting the need to achieve significantly lower exploration costs with the best possible project design. Still, the simultaneous management of distinct aspects may lead to the loss of focus and scatter of attention, resulting in increased costs, due to splitting functions.^4^ This aspect is unrelated with the issue of property, which is only relevant when there are difficulties associated with establishing contracts. If it is difficult to verify the quality of the infrastructure, it is stimulating to consider a PPP contract, since it is in the interest of the private sector that the project design powers the efficiency of clinical management.^4^ Without the presence of economies of scope between the two functions, and weighting in the considerable uncertainty regarding the production and the future outcomes, the private sector will not take its chances in clinical exploration, because this risk must be remunerated.

### 2.2 Models

Generally, healthcare PPPs are centred in hospitals that consume half of the budget defined for the health sector. In line with Abuzaineh et al.,^8^ there are essentially three types of models in this sector, namely:

1. *Model I*. Partnerships are created with the interest of the private party to design, build, finance, maintain, and/or operate the hospital infrastructure. These projects are classified as *greenfield* or *brownfield* if the structure is built from scratch or in case of restructuring, rehabilitation, and/or enlargement, respectively. In the end of the contracting period (25–30 years, typically), the assets revert to the State, which funds them during that period according to the availability of the contracted services and their performance.
2. *Model II*. It focuses on the delivery of discrete clinical services by the private party, which is responsible for financing the capital cost, maintaining the infrastructure, and operating and delivering specific clinical services.
3. *Model III*. The integrated PPP model is the most complex of all healthcare PPP models. It goes beyond improving or expanding hospital infrastructure, since it leverages the expertise of the private party to deliver and manage inpatient and outpatient services, essentially combining the main features of Model I and Model II.

The adoption of Model I, whether in hospitals or not, has become a common practice in many European Union countries, such as France, Greece, Germany, Italy, and Ireland (Spain and Portugal are some of the exceptions, since they have adopted Model I and Model III, and exclusively Model II, respectively^5^), after it emerged in the 1990s in the United Kingdom under the Private Finance Initiative (PFI).^8^ In particular, more than 100 hospitals were built through the PFI with a view to increase overall levels of service and lead to more investment without jeopardising the public treasury.^9^ The British PFI model can be considered as a subset of Model I, given its use of private sector debt and equity to deliver public services.^10^ Still, both models rely on a flexible legal framework^11^ to accommodate national and local specificities,^12^ without which there may arise legal and regulatory barriers to effective PPP governance.^13^, ^14^ In essence, Model I, also known as the infrastructure‐based model, is now the most common form of healthcare PPP; it has been implemented on a global scale by countries as varied as Australia, Canada, Egypt, Japan, and South Africa, because clinical management is not a responsibility of the private party, which leaves the core business of the hospital in the public sphere.^15^

Moreover, regarding Model III, the paramount example found in Valencia, Spain, must be addressed. In fact, the ambitious project behind the Alzira hospital sought to include both primary healthcare and referral clinics in the health district concerning the PPP contract. This allowed the private party to control the entirety of the health services for the target population, in an attempt to increase efficiency and promote more comprehensive health strategies. However, after almost 2 decades, what was once known as the Alzira model became recently under direct public management after financial issues, governance failures, and politics contributed to the termination of its contract.^16^

## 3 PORTUGAL AND ITS HEALTHCARE PPPs

### 3.1 State of the art

It was with the resolution of the Council of Ministers (RCM, from the Portuguese abbreviation of *Resolução do Conselho de Ministros*) no. 162/2001, of 16 November, that a new legal regime destined to the regulation of the creation and functioning of PPPs in healthcare was established. According to the same RCM, the Mission Structure of Health Partnerships (EMPS, from the Portuguese abbreviation of *Estrutura de Missão Parcerias Saúde*) was created, whose mandate would be renovated by the RCM no. 102/2004, of 21 July. EMPS is a core segment of the current Health System Central Administration (ACSS, from the Portuguese abbreviation of *Administração Central do Sistema de Saúde*) since 2011. Nevertheless, EMPS seeks to identify viable PPP projects in the health sector, as well as its respective launch and monitoring, based on national and international experiences. Accordingly, it is the responsibility of the ACSS to coordinate and monitor the enforcement of PPP contracts, with the inherent nomination of the contract manager, which intends to ensure the effective supervision of those contracts and the consolidation of the information by the private sector.^5^ On the side of the Ministry of Finance, the control is assured by Parpública, since the PPP process is, in the case of healthcare, under the tutelage of the Ministry of Finance and the Ministry of Health.

In 2002, the Decree Law (DL) no. 185/2002, of 20 August, was published, but was altered by the general PPP regime established by DL no. 86/2003, of 26 April, and DL 141/2006, of 27 July. The first one defines the PPP‐specific legal regime in healthcare, consigning the principles and instruments of this type of partnership in this sector, as a regime of private management and funding, between the Ministry of Health or institutions/services integrated in the SNS and private partners, for profit or not. In particular, and in what concerns risk sharing, this DL states that the distribution of risk must be made according to the capability of the intervening parties in managing risk, in line with Oudot.^17^ Additionally, it defines that the provision of healthcare can be primary, secondary, or continuous, or even as a support to its delivery.

Furthermore, the Regulatory Decree (RD) no. 10/2003, of 28 April, and RD no. 14/2003, of 30 June, came to approve the general terms of the proceedings previous to the celebration of management contracts to the establishment of PPPs in healthcare and the specifications of the management contracts that involved activities ranging from the conception to the exploration of the hospital.^18^ According to Simões,^15^ the aforementioned legal framework is quite flexible and allows the design of several contracting configurations. Given the multiplicity of possible formats, it was necessary to define a legislative methodology of each type of specification, thus establishing the most adequate partnership model and the respective proceeding previous to the contracting.

In 2001, the first wave of hospitals in a Model II PPP regime was announced, among which are included the hospitals of Sintra, Cascais, Braga, Vila Franca de Xira, and Loures (the first two are new hospitals and the last three are replacement hospitals), although the first one never came to fruition. Furthermore, this wave involved the conception, construction, funding, maintenance and exploration of the hospital building, and the provision of care by a consortium of two managing entities, namely:

1. One responsible for the management of soft facilities, that is, clinical management, with a 10‐year contract.
2. One responsible for the management of hard facilities, that is, building management, with a 30‐year contract.

Moreover, international experience dictates that countries, whose health system is mostly tax‐financed, tend to use PPPs more often, because they are a substitute for the need of public investment.^19^ Besides, in Portugal, healthcare PPPs seek solely to renovate the hospital set, but, abroad, the existence of PPPs is common in other areas, for example, health insurance in Austria, nursing homes in France, and specialised equipment units in Germany.

### 3.2 Supervising entities

Among the public entities responsible for regulating the performance of PPPs, the following stand out due to their pertinence: the permanent monitoring committee, the joint committee, the EMPS (extinct and replaced by the Technical Unit of Project Monitoring after the DL no. 111/2012, of 23 May), the user deputy or ombudsman, the Health Regulatory Entity , the Constitutional Court, the Assembly of the Republic and the Court of Auditors.^20^

Due to the lack of know‐how in the public sector human resources and the high complexity inherent to contracting in a PPP regime, private consulting entities should intervene in the process of designing and managing these projects. Nonetheless, resorting to such entities is merely punctual, which reveals a serious gap in the development and monitoring of a PPP project.^21^

### 3.3 Procedural lethargy

A crucial factor in PPP contracts is found in costs, whether monetary or time, associated to the creation of the hospital, since the beginning of the public tender until the signing of the contract. Table 1 shows the slowness of the process with data from Tribunal de Contas.^22^

**TABLE 1:** Time due (in days) between the start date and the signing of the contract for each Portuguese first‐wave hospital in a public–private partnership regime

	Cascais	Braga	Vila Franca de Xira	Loures
Start date	13 May 2004	18 Nov 2004	20 Apr 2005	12 May 2006
Preparation and previous evaluation	245	76	32	139
Approval and launch	66	40	30	88
Proposal presentation	159	138	226	136
Proposal evaluation	404	509	436	690
Competitive negotiation	‐	171	380	132
Final negotiation	‐	315	‐	‐
Start of activity	Feb 2012	Jan 2009	May 2011	Jul 2011

Between the start date and the start of activity, at least 5 years go by. Still, this is not an unusual situation, as Ahadzi and Bowles^23^ and Pollock et al.^24^ state regarding the international experience in the delay and high costs of PPP hospital construction projects.

### 3.4 Inclusion of clinical activity

Based on Hart's^25^ model, Barros^19^ and Campos and Simões^18^ claim that the inclusion of clinical management in a PPP contract must bear in mind the vulnerability of social benefits to insusceptible investments and the equality of the public cost of those investments between the public and private sectors. However, according to Silva,^26^ it is not advisable given the difficulty in measuring clinical activity and in identifying and managing public services' risks. Still, the highest potential efficiency gains associated to private management may make the inclusion in the private sector beneficial, and the PPP contracts include an extensive list of quality indicators, which reduces the concern with those externalities.^19^ However, the issue regarding the incapacity to correctly assess clinical activity becomes more relevant in the case of hospitals that treat more complex users, especially when coupled with the inexperience of the PPPs involving the exploration of the clinical activity^26^ and the frequent need to renegotiate, since it is impossible to accurately predict technological evolution.^19^

Moreover, Barros^19^ claims that noncontractible investments have two potential impacts: the increase of partnership benefits and lower infrastructure and operating costs. A decreased State investment in these areas yields greater benefits and/or lower costs, thus assuring that such investment is efficiently conducted is an issue to be solved by the partnership. Therefore, there is an economic problem associated to the definition of the incentives regarding non‐contractible investments, being those usually made indirectly. Hence, through relevant decisions and the capacity of monitoring performance, the interest of including or not the clinical exploration in PPPs varies. Additionally, one also needs to consider that non‐contractible investments depend on the (re)negotiation skills of both parties. Without indirect incentives to such investments, regulating the renegotiation procedure would contain exclusively the price of new investments and the replacement costs. Nevertheless, since new investments are typically focused on technology, and given the current overview of technological development, the private sector has a higher bargaining power than the public one, which translates into negative impacts for the public treasury. According to Barros,^19^ these effects are reinforced when clinical exploration is owned by the private sector. For this reason, there is more than one PPP model in healthcare. Bottom line, hospitals with higher technological complexity should exclude clinical exploration from their PPP contracts and adopt Model I, and hospitals with no significant technological changes over time may opt to include clinical exploration in the PPP contract and adopt either Model II or Model III.

### 3.5 Market access

Resorting to a PPP model assumes that both the project and the selected proposals are advantageous to the eyes of public interest. As such, the private partner should be selected based on a transparent and competitive public tender with at least one proposal presenting a cost inferior to the public sector comparator (PSC). The public tender is traditionally long, complex, and costly for both parties, and comprises eight phases^15^: noticing, proposal presentation, public act, qualification, proposal selection, negotiation, adjudication, and contract drafting.

First‐wave hospitals' public tenders were all launched between 2003 and 2005, and 4–6 private consortiums applied. Among them, José de Mello Saúde was the private party that won two out of the four tenders, namely, Braga and Vila Franca de Xira, with Hospitais Privados Portugueses winning the tender for Cascais. However, the public tender for Loures was annulled due to critical flaws in the evaluation of the proposals, but a new tender was created about 3 years later—this time, only two proposals were submitted, with Espírito Santo Saúde being chosen as the winner. Note that the hospitals took approximately 4–7 years since the selection of the winner to be operational. Besides, one verifies that the same four consortiums applied to all public tenders, denoting the attempt to achieve a monopoly of private healthcare in the Portuguese market.

On another angle, the documents submitted to the contracting public entity and the qualifying criteria can be found in the 4th chapter of the proceedings programme: articles 13th and 31st for Loures, and articles 15th and 33rd for Cascais, Braga and Vila Franca de Xira. On the one hand, the required documents were absurdly detailed and numerous, which impacted the triage and evaluation by the public entity. Cabaço^27^ also notes that the specifications were inadequate to the PPP model, denoting a misalignment with the requirements. On the other hand, the criteria attempted to assess the competence of each contestant, but their qualitative nature made a fitting measurement impossible. As Carias^28^ points out, such criteria should be quantified, including the number of accomplished projects and the experience in funding amounts above a certain value.

Moreover, the selection of the consortiums was focused on criteria *price* (limited above by the PSC) and *technical quality* of their respective proposals. Still, the level of criteria detail was either too high or too low, which prevented the comparison of proposals. Consequently, the expected duration of this phase had discrepancies of over 220%, since the predicted 5–6 months oscillated between 1 month and 2 years in reality. Afterwards, the two best proposals were chosen and were given the freedom to alter their initial proposal to a final price not inferior to the initial one. Nonetheless, the final negotiation with the winner was conducted a posteriori with no price alterations.

### 3.6 Risk sharing

One of the main aspects to consider when formulating a PPP is risk, whether it is related with construction, exploration, supply, contract management, legislation, finances, technology or politics.^5^ DL no. 185/2002, of 20 August, mentions that risk must be attributed to the parties that are more competent in managing it. Thus, two hypotheses can be established: one of the partners owns all risks, or risk is allocated equally or not to both partners. Since the State has a greater capability to withstand risk, the private sector needs to have a higher risk management capability, in order to generate mutual benefits.

Typically, risks are categorised by a PPP's life cycle. Table 2 sums up the risk sharing of the first‐wave PPP hospitals in Portugal.

**TABLE 2:** Risk sharing of the first‐wave Portuguese healthcare public–private partnerships, adapted from Cruz and Marques^5^

	Cascais	Braga	Vila Franca de Xira	Loures
Construction	Mostly assumed by the private party.			
Exploration	Assumed by the private party. The State assumes part of the risk associated to the early payment of the remuneration.	Assumed by the private party. The State assumes part of the risk associated to the transition of databases' ownership, the clearance of compensations, and the costs of emergency services.		Assumed by the private party. The State assumes part of the risk associated to the early payment of the remuneration and the costs of emergency services.
Supply	Mostly assumed by the private party. The State assumes the risk related to the supply of users outside of the hospital's geographical area of influence.			
Contract management	Assumed by the State.			
Asset ownership	Mostly assumed by the State.	Shared between the State and the private party.		

It can be observed that there are substantial differences regarding risk allocation, but there is a tendency in the attempt to allocate more risk to the private sector, especially in more recent contracts. Furthermore, the risk allocation between the four hospitals is quite similar, apart from Cascais, which can be explained due to it being the first PPP experience in healthcare in the country. Finally, note that, globally risk sharing is in line with the international guidelines.

For the sake of mitigating such risks, Carias^28^ proposed a series of strategies consisting in the establishment of factors and criteria to minimise their consequences at a pre‐contractual level, a conceptual/construction level, and an operational level.

Nevertheless, the private partner is responsible for the activities developed under the scope of the contract (e.g., obtaining licences and authorisations, and implementing systems that monitor quality and performance), while the public partner inspects the former's activities. The risk of asset seizure, redemption or termination, foreseen in the management contract, placed a significant toll on the private partner, since those risks are associated to a poor performance of one of the two managing entities. This performance can eventually be corrected before the intervention of the public entity.

### 3.7 Contract renegotiations

Attributing concession contracts to private entities by the State has been revealing the trend of a high number of contract renegotiations, with profound alterations of some initially contracted clauses, which can result in additional charges for the State, since they are carried out in a non‐competitive environment. Even though renegotiations may have a positive effect on both parties, their infamous character is notoriously acknowledged. Note that such renegotiations may come from both parties, the purpose of which being more or less transparent/beneficial to society and the common good.

If there is no possibility to specify all actions to be taken by the partners in the contract, one has to accept the existence of renegotiations as a natural consequence of PPPs. Particularly, risk corresponds to situations whose probability of occurring is foreseen and is known by both parties, thus it is uncomplicated to insert in the contract. Still, there is a degree of uncertainty surrounding risk that prevents contracting certain clauses that indicate actions to be taken when the scenario is unknown, especially in healthcare, where contracts are long term.

### 3.8 Contract management

The most important and long‐lasting phase of a contract in a PPP regime is contract management between the State and the private partner. This is where the essence of the contract lies. If the public entity wants to collect all of the benefits expected from the partnership, it is important to pro‐actively ensure the monitoring of the contract enforcement by the State, which assumes even more importance, since it is celebrated in the socially relevant healthcare sector.

Indeed, the contract manager is chosen via a public tender and assigned by the Ministry of Health. According to the article 61st of the RD no. 14/2003, of 30 June, a permanent monitoring committee designated by the public entity must be assigned to its respective hospital unit in a PPP regime. This committee acts as an in loco extension of the public entity, being desirable that its core is comprised by elements of the public entity responsible for formulating and renegotiating the contract. Among its competencies, verifying the compliance with obligations of the managing entities, ensuring the daily connection between the two entities, and drafting periodical reports on hospital activity are highlighted. Consequently, this committee must have unrestricted and continuous access to all documentations related to operations intrinsic to the PPP contract. This implies a complex, dense, and demanding relation, thus article 69th of the aforementioned RD mentions the creation of a triangularly composed committee, which shares the same rights as its counterpart, but intervenes in the elaboration of proposals whose adoption is translated in the modification of the management contract or its terms of enforcement.^15^

The evaluation of clinical and non‐clinical performances in the relation between the PPP operator and the user is foreseen in article 70th of RD no. 14/2003, of 30 June, with the creation of a user deputy that collects all complaints and suggestions of the user. This deputy must draft recommendations and ease the decision‐making of the managing entities in the sense of correcting the detected flaws. Moreover, article 68th of the previously mentioned RD refers the regular and periodical performance evaluation of the managing entities on the dates established in the contract, with the purpose of assessing if the contract is renewable or not (article 23rd of the same RD). The reports and accounts that the private managing entity are obliged to draft should also be taken into account in the performance evaluation. It is the Ministry of Finance that regulates and controls the enforcement of such partnership at an economic–financial level, leaving the remaining subjects to entities designated by the Ministry of Health.

Moreover, creating a monitoring system in pursuance of satisfying three core goals is necessary. These objectives consist on allowing the recording of collected information and, thus, a self‐evaluation of the managing entities, recording expected and observed performance parameters to ease their comparison and detect flaws, and facilitating the evaluation of the managing entities by contracting public entity. The performance parameters common to the management contracts of all first‐wave PPP hospitals comprehend a set of 52 distinct indicators.

### 3.9 External evaluation of the PPP experience

As a public interest protector, the Court of Auditors has advisory, preventive supervision, and judicial inspection functions. If conducted by entities organically inserted in the State's structure or politically, judicially, and technically independent instances, the financial control can be internal or external, respectively. Hence, the Court of Auditors exerts an external control over PPPs in order that these behave within the boundaries of the State's budgeting capacity.^20^

In 2006, the total expenditure of the healthcare PPPs ascended to 122 M€ coming almost exclusively from the 1990s unsuccessful PPP pilot experiment of the Amadora‐Sintra hospital with 117.2 M€, although the EMPS originated, alone, 4.8 M€. According to Tribunal de Contas,^29^ the expenditure with PPPs between 2007 and 2038 was predicted to be 5534.8 M€, which included a considerable increase in public expenditure between 2007 and 2016, and a decrease from then on to 2020.

In 2008, the first monitoring report of the ACSS stated that PPPs 'are yet to reflect in a true and appropriate way the financial position and the results of the operations of the whole universe of entities that integrate the SNS, harming the integrity, accuracy, and transparency of the information report on the financial‐economic situation of the SNS'. This reveals strong gaps in the task of auditing and enforcing the contract by the responsible entities. In fact, without an effective control, it is not possible to confirm the potential gains in terms of sustainability of the legal–contractual model. Hence, the Court of Auditors proposed several recommendations in this regard, scilicet:

1. There is the need that the available information on the accounts reflects in a true and appropriate way the financial situation and the results of all the healthcare‐providing entities of the SNS.
2. All the information on the financial–economic situation of the SNSs' entities provided by the various governmental organisms must be reliable, accurate, rigorous, and transparent.

Later, Tribunal de Contas^22^ issued a rather critical appraisal regarding the whole PPP contract development process in terms of sectorial planning, the assessment of previous capabilities, the intervention of the competent public entities, public coherence, the model, the project flow, public tender components, procedural design, proposal assessment, resources, and the capacity of public management response.

As we have been discussing so far, the lack of experience of the Ministry of Health constituted a serious problem on the development of this type of projects. Without any type of international benchmark, an experimentalist and innovative approach was opted for, but with no actual pilot experiment, which came to be a key factor for the slippage of PPP projects.^28^ If, on the one hand, such experiments increased the know‐how of the public entities responsible for the SNS and its sustainability, on the other hand, that experience is characterised by successive model changes. It was clear that the State did not have a well‐defined strategy nor well‐established goals. One of the more pressing examples was the annulment of the public tender for the Loures hospital. Furthermore, the lack of synergy alignment between the potential partners regarding service standards and their technical support was reflected on the procedural lethargy and on the inability to properly evaluate the different proposals. For instance, in 2007, four simultaneous public tenders were still being held, which resulted in an increase in the efforts of the private parties to match the specifications of each tender. However, these efforts were not matched by the public sector, demonstrating a profound amateurism of the governing bodies and the exclusively political drive behind the first healthcare PPP vacancy. In fact, Tribunal de Contas^30^ pointed out that 'the auditing has, in many cases, pinpointed performance flaws of these projects, some of them serious enough to the point of being in conditions of contract termination by the State'. This report also highlighted that, in 2012, the efficiency of the expenses incurred by the State with healthcare PPPs was not objectively assessed. The heavy bill of the State concerning healthcare PPPs is equally verified by the Direção‐Geral do Tesouro e Finanças^31^ given the increase in illiquid expenses over 44% on the second trimester of 2012, when compared with its homologous period. Regarding clinical management contracts, the Court of Auditors concluded that they were more onerous than expected, but their transfer to the public sphere would lead to efficiency losses, thus proposing the extension of the contract for another 10 years or the attribution of the clinical management to another private party.

## 4 CHALLENGES, OBSTACLES, AND OPPORTUNITIES OF THE CURRENT MODEL

Developing a project in a PPP regime has advantages and disadvantages. Maximising the social value is the ultimate goal of these types of endeavours, thus it is relevant to mitigate the disadvantages and make the most of the advantages. In general, a PPP project allows the integration of its several phases. Since those projects are highly complex and require high investments, separating the different phases in an outsourcing regime with different partners would lead not only to an increase in bureaucracy, but also to higher costs and unattributed responsibilities. For this reason, integrating all of these phases allows attributing construction and maintenance responsibilities to a single (private) contractor, ensuring innovative solutions in the design phase, given the competition among different consortiums. This competition is as higher as the market in which the project is inserted is attractive—this is the case of healthcare, due to its social value and business volume. Besides, the mechanism of fixing a construction price places the risk of budget slippages entirely on the private party, who seeks to minimise expenses and maximise profit. Hence, it is possible to allocate spare public resources in sector where its scarcity is more notorious and severe. For instance, it is possible to capture synergies with the vertical integration of clinical management and infrastructure management (something that characterises the first wave of PPP hospitals). Still, it is necessary that the State takes responsibility on auditing and regulating the functioning of PPPs, in order to avoid moral hazard and mismanagement of the private sector and protect public interest.

However, there are plenty of disadvantages in this partnership model. If it is true that there may be a release of additional public resources due to the private investment, it is also true that private funding is more expensive given the nature of risk. Since public expenditure does not count for computing the public deficit, it escapes budgeting control, that is, there is a *debudgetisation*. There is also a great difficulty in predicting long‐term scenarios, because technologies and medical procedures are constantly changing. Since the contract should contain as many predictions as possible, these are not entirely trustworthy. Hence, renegotiations are needed in face of the lack of competition and significant information asymmetry. This reveals the fragility of the PPP model in four aspects: decrease in social welfare, increase in State expenses, increase in tariffs, and change in risk sharing. Thus, an efficient and effective management and contract regulation become essential, despite the frequent diversion of both the regulator and the managing entity by interest groups.

In general, the success of the PPP model in healthcare depends on improvement measures regarding the:

1. Decision‐making process associated to the best contracting model for a certain project.
2. Budgetary treatment, since PPP expenses are not accounted for purposes of computing the public debt deficit.
3. Transparency, accountability, and participation.
4. Creation of public and apolitical agencies with judicial and administrative autonomy, and equipped with fundamental tools for a proper and efficient management of the process.
5. Effective contract management.
6. Increase of the State's bargaining power, minimising information asymmetries and inadequate termination clauses.

Thus, ensuring the success of a PPP necessarily includes evaluating the performance of the State and its private partner, which monitors service quality and the fulfilment of the contracted goals. From the start, an appropriate set of performance indicators must be established, given the available circumstances and information system.^32^ This set should be as exhaustive as possible. Besides, it is necessary to manage inter‐entity relations, proceeding and reinforcing communication, managing stakeholders, ensuring their involvement in several committees, and managing strategic relations with the financers.^33^ Knowing how to manage change, the private partner's complaints, the factors inherent to attributing functions, the alterations of output specifications, automatic contractual changes, the demands of the service, and technological advancements are other key factors for the success of this partnership. Still, integrating mechanisms that allow to deal with and manage conflicts is vital, as well as to ensure the maintenance and revision of contingency plans and their response in case of disaster.

Finally, it is not feasible a real partnership without an institutional environment of trust among partners. Consequently, an aspect that needs to be corrected is the weak collaboration between the two parties, which translate into defensive behaviours that prevent a solid cooperation and efficiency gains, deviating PPPs from a partnership concept and approximating them to a concession concept.

## 5 CONCLUSION

If the public perception surrounding the concept of healthcare PPPs is not positive (in Portugal, unlike in countries like China,^2^ Norway^3^, or Turkey^34^), that may be due to several factors that empowered and justified that idea. Even if the concept itself is interesting and potentially advantageous both to the public sphere and the private sector when it is well designed,^3^ well managed and well controlled, the practical results did not translate into value gains, especially to the public entity, harming public interest. In fact, some authors claim that healthcare PPPs are more technically efficient than traditionally managed institutions.^35^ Still, everything leads one to believe that the existence of healthcare PPP in Portugal was exclusively because of political reasons.

Starting with the difficulty in defining the private proposal that presented the best value for money, whether due to the undefinition and lack of quantification of criteria and weights of public tenders, or the adopted approach, which demanded a significant human resources' effort, since simultaneous public tenders were being held, the complexity and the lack of experience, clarity, and transparency in selecting the consortiums were quite clear in the market access phase.

Furthermore, including clinical management in the private sphere, although innovative and potentially inductive of efficiency and effectiveness gains, is not an advisable idea in healthcare, since this area is in constant change, which makes risk allocation of new investments demand renegotiations, leading to a compensation of efficiency gains by initially unpredicted extraordinary expenses. Besides, the inclusion of this aspect on the management contract increases the complexity of the public tender and the evaluation of proposals of the first wave of PPP hospitals.

The value for money of such a project is intrinsically linked to the risk incurred by each entity. Identifying, allocating, and measuring probabilities and their impact, and planning the minimisation of the latter are key factors to create value for money and, consequently, a stable relationship among partners. In general, risk sharing followed international guidelines, which results from the efforts towards drafting a management contract capable of ensuring the interests of both parties. Nevertheless, despite the usual proper construction of this contract, there are only a few times where it is well managed and monitored, due to undetected flaws, inopportunely unapplied penalties and renegotiations, which are nothing else but mechanisms that injure the State to the detriment of the profit‐seeking private sector. One of the reasons lies on the adulterated flow of users and undue referrals to other hospitals or other facilities in the continued care network in the attempt of avoiding pathologically more complex users (users who consume more resources) or extra users (users that exceed supply predictions). Another reason is in the difficult access to monitoring systems. Bottom line, the inexperience of the State regarding its incapacity to adequately oversee management contracts is highlighted.

Perhaps, the most compelling flaw is the lack of specification concerning mechanisms that delimit renegotiations. In healthcare, it is impossible that no renegotiations occur during the contract management phase, given the inherent features of this sector and the wide time horizon that these partnerships deal with. Nonetheless, one may attempt to minimise those renegotiations to an absolute and relevant minimum, in order to maintain the alignment between public and private interests, which is, in the end, the purpose of a PPP.

## CONFLICT OF INTEREST

Miguel Alves Pereira, Diogo Cunha Ferreira and Rui Cunha Marques received research grant PTDC/EGE‐OGE/30546/2017 from FCT. Miguel Alves Pereira also received research grant SFRH/BD/149283/2019 from FCT.

## ETHICS STATEMENT

The current study did not require ethical approval, since it did not involve the participation of human and/or animal subjects.
